# Advances in Pure Drug Self-Assembled Nanosystems: A Novel Strategy for Combined Cancer Therapy

**DOI:** 10.3390/pharmaceutics17010068

**Published:** 2025-01-06

**Authors:** Runyan Niu, Xuexue Liu, Xian Yang, Xiao Du, Siliang Wang, Xiaolong Ma, Shaoping Yin, Lihua Shao, Jinping Zhang

**Affiliations:** 1 Nanjing Medical Center for Clinical Pharmacy, Department of Pharmacy, Nanjing Drum Tower Hospital, Affiliated Hospital of Medical School, Nanjing University, Nanjing 210008, China; nry20210910@163.com (R.N.); yangxian-gp4@163.com (X.Y.); duxiaojianai@126.com (X.D.); wsl_dth@126.com (S.W.); 2 Department of Pharmacy, Nanjing Drum Tower Hospital, School of Basic Medicine and Clinical Pharmacy, China Pharmaceutical University, Nanjing 210008, China; 3 Department of Hematology, Nanjing Drum Tower Hospital Clinical College of Nanjing University of Chinese Medicine, Nanjing 210008, China; 15037306560@163.com; 4 Department of Colorectal Surgery, Nanjing Drum Tower Hospital, Affiliated Hospital of Medical School, Nanjing University, Nanjing 210008, China; qdumaxiaolong@126.com; 5 School of Pharmacy, Nanjing University of Chinese Medicine, Nanjing 210008, China; ysp0305@126.com

**Keywords:** nanosystems, self-assembly, carrier-free, combination treatment, cancer therapy, pure drug

## Abstract

Nanoparticle-based drug delivery systems hold great promise for improving the effectiveness of anti-tumor therapies. However, their clinical translation remains hindered by several significant challenges, including intricate preparation processes, limited drug loading capacity, and concerns regarding potential toxicity. In this context, pure drug-assembled nanosystems (PDANSs) have emerged as a promising alternative, attracting extensive research interest due to their simple preparation methods, high drug loading efficiency, and suitability for large-scale industrial production. This innovative approach presents new opportunities to enhance both the safety and therapeutic efficacy of cancer treatments. This review comprehensively explores recent progress in the application of PDANSs for cancer therapy. It begins by detailing the self-assembly mechanisms and fundamental principles underlying PDANS formation. The discussion then advances to strategies for assembling single pure drug nanoparticles, as well as the co-assembly of multiple drugs. Subsequently, the review addresses the therapeutic potential of PDANSs in combination treatment modalities, encompassing diagnostic and therapeutic applications. These include combinations of chemotherapeutic agents, phototherapeutic approaches, the integration of chemotherapy with phototherapy, and the synergistic use of immunotherapy with other treatment methods. Finally, the review highlights the potential of PDANSs in advancing tumor therapy and their prospects for clinical application, providing key insights for future research aimed at optimizing this technology and broadening its utility in cancer treatment.

## 1. Introduction

Cancer is one of the most significant threats to human life. The estimates suggest that approximately one in five men or women develop cancer in a lifetime, whereas around one in nine men and one in 12 women die from it [1]. Among the various cancer treatments currently applied in clinical practice—including conventional therapies such as surgery, chemotherapy, and radiotherapy, as well as innovative treatments like phototherapy, immunotherapy, and gene therapy—chemotherapy remains a cornerstone [2,3]. As a systemic treatment, chemotherapy can reduce tumor size before surgery or radiotherapy and eliminate metastatic cancer cells due to the widespread distribution of anticancer drugs in the bloodstream [4,5]. Nonetheless, chemotherapy has several limitations. Traditional chemotherapeutic drugs are known to distribute non-specifically within the body, potentially causing severe side effects during treatment. Additionally, the phenomenon of multidrug resistance (MDR) that arises after repeated use of the same chemotherapeutic agents can significantly reduce the effectiveness of chemotherapy [6]. Therefore, it is essential to develop more effective strategies to enhance the efficacy of chemotherapy, maximizing its benefits while circumventing its disadvantages.

Utilizing drugs as carriers for delivering therapeutic agents represents an innovative and promising strategy to enhance cancer treatment efficacy. This approach offers a key advantage; the drug carrier can significantly prolong the agent’s circulation time in the bloodstream and enhance its therapeutic effect while simultaneously reducing adverse side effects [7]. Recent advancements in nanotechnology have greatly accelerated the development and application of various nanocarrier formulations, which have become integral to the field of cancer therapy. These nanoformulations include liposomes, micelles, albumin nanoparticles, polymer nanoparticles, and inorganic nanoparticles [8]. To date, the U.S. Food and Drug Administration (FDA) has approved over 60 nanomedicines, more than half (53%) of which are specifically intended for cancer treatment. Several of these nanomedicines have already progressed to clinical application [9]. For example, in 1995, the FDA approved the first lipid nanoparticle formulation, Doxil (PEGylated doxorubicin liposomes). This formulation has demonstrated superior efficacy and reduced cardiotoxicity in treating metastatic ovarian cancer and Kaposi’s sarcoma [10]. Over the past few decades, the development of nanotechnology has led to the research and application of various nanoformulations in cancer therapy, including liposomes, micelles, albumin nanoparticles, polymer nanoparticles, and inorganic nanoparticles [8]. For example, albumin-bound paclitaxel (Abraxane) has demonstrated superior therapeutic efficacy and better tolerability compared to paclitaxel by improving its pharmacokinetic properties [11].

Despite the advantages of carrier-assisted nanomedicines in cancer treatment, several limitations persist. Traditional nanodrug delivery systems exhibit notable drawbacks, such as complex preparation processes, high production costs, low drug loading capacity, and potential nanomaterial-related toxicity. These issues have constrained their clinical application [12].

Pure drug-assembled nanosystems (PDANSs) represent an emerging research direction. These systems are composed entirely of pharmacologically active compounds, significantly reducing the need for non-therapeutic excipients [13]. Compared to traditional nanodrug delivery systems, PDANSs offer several advantages, including high drug loading efficiency and simplified formulation processes (Table 1) [14,15,16]. However, there are notable distinctions between them. Firstly, PDANSs can be constructed using both chemical drugs and certain biological agents, such as antibodies, proteins [17,18], and nucleic acids [19]. Secondly, PDANSs are typically smaller in size than conventional drug delivery systems, which allows them to exploit the enhanced permeability and retention (EPR) effect, thereby promoting drug accumulation within tumor tissues. This advantage helps carrier-free nanomedicines overcome multiple stromal barriers, enhancing tumor penetration and drug perfusion capabilities [20]. Additionally, the preparation of PDANSs avoids the use of extra carrier materials, not only improving drug loading efficiency (with drug nanoparticles achieving drug loading rates as high as 100%) but also alleviating concerns regarding the biosafety of carrier materials, such as toxicity and immunogenicity. Finally, PDANSs can be produced through simple methods without the need for high-energy machinery (e.g., high-pressure homogenizers), facilitating large-scale manufacturing [21,22]. Overall, PDANSs are considered a promising strategy for future clinical applications.

**Figure 1:**
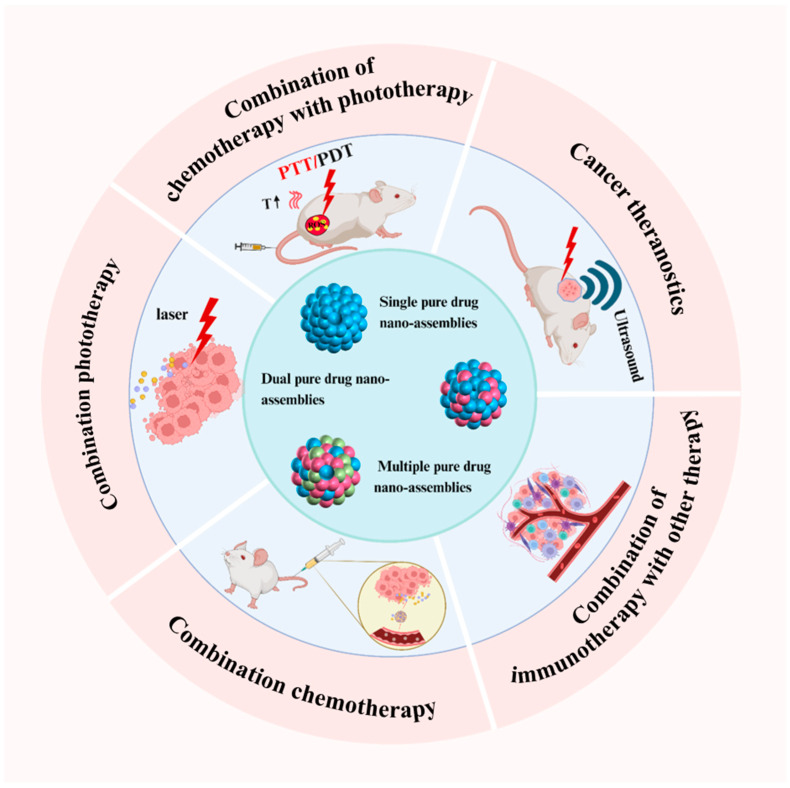
Schematic overview of the significant application advancements of pure drug-assembled nanosystems in tumor therapy, including cancer theranostics, combination chemotherapy, combination phototherapy, combination chemotherapy with phototherapy, and the combination of immunotherapy with other therapy. Created with BioRender.com (accessed on 20 November 2024).

This review presents a detailed overview of recent progress in the application of PDANSs for cancer therapy. It begins by exploring the self-assembly mechanisms and fundamental principles underlying the formation of PDANSs. The discussion then progresses to strategies for the nano-assembly of single pure drugs and the coordinated assembly of multiple pure drugs. Subsequently, the review evaluates the therapeutic efficacy of combination approaches, leveraging pure drug nano-assemblies, encompassing both diagnostic and therapeutic applications. These therapeutic strategies include combinations of chemotherapeutic agents, phototherapeutic methods, integrated chemophototherapy, and the integration of immunotherapy with other treatment modalities. Finally, the review highlights the potential of PDANSs in advancing tumor therapies and discusses their prospects for clinical translation (illustrated in Figure 1). It offers valuable insights for future research aimed at optimizing PDANSs and broadening their application in cancer treatment.

## 2. Mechanisms of Pure Drug Self-Assembly

**Figure 2:**
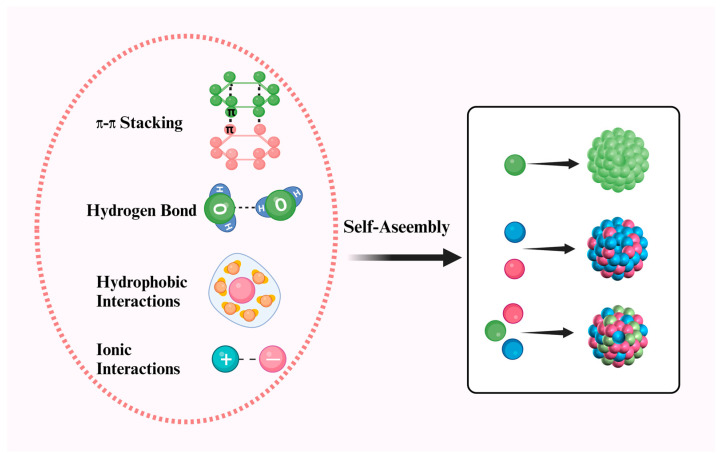
Schematic illustration of the self-assembly mechanism of pure drugs. The self-assembly of pure drugs into nanoparticles is primarily driven by non-covalent interactions, including hydrogen bonding, π-π stacking, and hydrophobic interactions. For example, photosensitizers such as chlorin e6 (Ce6) and mitoxantrone, which feature aromatic conjugated structures and an abundance of oxygen and nitrogen atoms, can self-assemble into stable nanoparticles through these interactions. When combined with 660 nm laser irradiation, these nanoparticles enable non-invasive tumor ablation [38]. Similarly, natural compounds like magnolia bark form stable, soluble, carrier-free nanoparticles through hydrogen bonding and hydrophobic interactions, effectively enhancing tumor targeting and therapeutic efficacy [39]. Furthermore, Xiao et al. engineered DOX-PhA nanoparticles via electrostatic self-assembly, leveraging the interaction between the -NH^2+^ group of doxorubicin and the -COO^−^ group of pheophorbide A. This design facilitated a synergistic approach for combined photo/chemotherapy in cancer treatment [40]. A growing body of evidence highlights that π-π stacking, hydrogen bonding, and hydrophobic interactions are key mechanisms underlying the self-assembly of PDANSs. These mechanisms offer a robust and safe framework for the rational design of clinical drug formulations, presenting a promising strategy to enhance therapeutic outcomes. Created with BioRender.com (accessed on 20 December 2024).

During the self-assembly process of anticancer drugs, the most commonly used method is the one-step reprecipitation technique. Typically, the drug is dissolved in an organic solvent and then added dropwise into water under vigorous stirring. Due to solvent exchange effects and intermolecular interactions among drug molecules, nano-assemblies form spontaneously [23]. Functional groups in the chemical structure of anticancer drug molecules induce intermolecular interactions between the drug molecules themselves or between the drug and water molecules. Generally, drug molecules with the following structural features are more likely to be good candidates for PDANSs: (a) abundant aromatic structures (driving π-π stacking interactions) [24,25]; (b) various ionic groups (driving ionic interactions) (Figure 2) [26,27]; and (c) rich oxygen and nitrogen atoms (driving hydrogen bonding and coordination) [28]. Typically, if the intermolecular interactions among drug molecules are dominant, the drug precipitates in an aqueous solution. Conversely, if the interactions between the drug and water are stronger, the drug dissolves in the aqueous solution. For example, DiR molecules have an aromatic head that provides strong π-π stacking forces for aggregation, while the long aliphatic tail introduces steric hindrance to prevent excessive aggregation [29]. Additionally, different morphologies of self-assembled nanodrugs exhibit varied biological effects. However, the self-assembly of single drugs can be challenging. For instance, ursolic acid (UA) can self-assemble into carrier-free nanodrugs with the aid of ultrasonication [30]. Self-assembly facilitated by other molecules can more easily balance intermolecular interactions. The extensive literature shows that photosensitizers such as indocyanine green (ICG) [31], chlorin e6 (Ce6) [32], and protoporphyrin IX (PPa) exhibit good co-assembly capabilities [33]. In one study, Ce6 was used as a template to co-assemble with various anticancer drugs, including DOX and HCPT [34,35], likely due to π-π stacking interactions among aromatic groups [36]. These strong π-π interactions allow different drug molecules to have good affinity and form aggregates through edge-to-face, offset, or face-to-face stacking. PDANSs typically require complex synthetic schemes that are difficult to predict, execute, and control. Fortunately, with advancements in computer science, molecular dynamics simulations have been employed to study the supramolecular interactions and carrier selection of nanoparticles. Shamay et al. designed a quantitative structure nanoparticle assembly prediction (QSNAP) model that accurately predicts the composition and particle size of stable tumor-targeting nanoparticles formed by the self-assembly of sulfated indocyanine dyes and hydrophobic drugs [37]. Although the application range of such techniques needs further expansion, we remain hopeful for the future development of broadly applicable technologies that can rationally predict and optimize PDANSs.

## 3. Pure Drug Molecule Nano-Assembly

PDANSs are entirely composed of active drug molecules, free from any inactive molecules or chemical substances. Based on the composition of the drug modules, PDANSs can be classified into three types, namely (1) single pure drug nano-assemblies, assembled from a single drug module; (2) dual pure drug nano-assemblies, co-assembled from two drug modules; and (3) multi-pure drug nano-assemblies, co-assembled from more than two drug modules. The assembly of these drug modules is primarily driven by non-covalent interactions, including hydrophobic interactions, intermolecular π-π stacking, hydrogen bonding, and electrostatic forces. For hydrophobic molecules, which have high surface free energy, a small amount of surfactant can be added to enhance colloidal stability. In this section, we will introduce and discuss these three types of PDANSs (Table 2).

### 3.1. Single-Drug Self-Assembly

**Figure 3:**
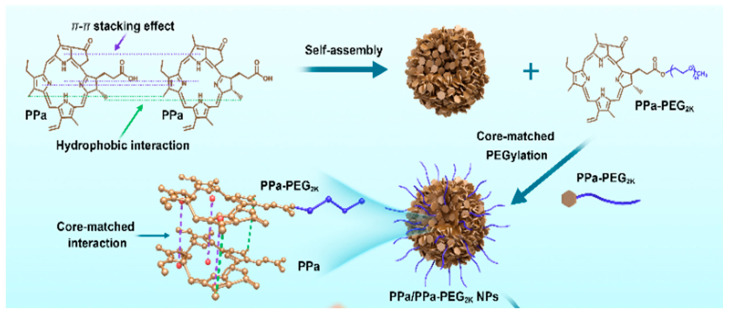
Schematic representation of self-assembly of PPa and efficient PDT under laser irradiation. Core-matched PPa nano-assembly was formed by modification of PPa-PEG2K [41].

Single-drug self-assembly for antitumor therapy primarily aims to enhance the physicochemical properties, biological characteristics, and pharmacokinetics of the drugs, leading to improved therapeutic efficacy. Notably, pure drug nano-assemblies formed from individual chemical or biological drugs offer a simpler preparation process and higher drug delivery efficiency, providing a more straightforward platform for carrier-free nanomedicines [14,48]. For instance, Zhang et al. developed a PEG-modified nano-assembly from pure photosensitizer PPa [41]. The PEG2K-modified PPa nano-assembly, stabilized by π-π stacking and hydrophobic interactions between the PPa core and PPa-PEG2K shell, exhibited excellent colloidal stability (Figure 3). Compared to PCL-PEG2K of a similar molecular weight, PPa-PEG2K achieved a high drug loading capacity of 74.8%, demonstrating significant advantages in cellular uptake, tumor homing, and pharmacokinetics. This facilitated rapid accumulation at the tumor site, ultimately enhancing antitumor efficacy in BALB/c mice bearing 4T1 xenograft tumors. Additionally, dihydroartemisinin (DHA), a natural product isolated from Artemisia annua, has shown effective tumor inhibition through various mechanisms [49]. However, the clinical application of DHA is limited by its poor water solubility and bioavailability. Li et al. and colleagues developed carrier-free nanoparticles (DHA NPs) based on DHA self-assembly [42]. These nanoparticles, formed through hydrogen bonding and hydrophobic interactions, exhibited near-spherical morphology, narrow size distribution, high drug encapsulation efficiency (>92%), and good stability. Both in vitro and in vivo studies demonstrated that DHA NPs had significantly superior antitumor efficacy compared to equivalent doses of DHA. Despite these advantages, most antitumor drug molecules do not inherently possess strong self-assembly capabilities, necessitating specialized preparation methods such as precise temperature control, appropriate concentration, and ultrasonic treatment. Even with these methods, excessive crystallization can lead to overly large spherical or rod-shaped particles [50].

### 3.2. Co-Assembly of Dual Drugs

The co-assembly of two distinct drug molecules can be described as the equilibrium of interactions between the two molecules. Compared to single-drug assemblies, dual-drug co-assemblies exhibit significant advantages in assembly capabilities. For instance, individual paclitaxel (PTX) self-assembles into unstable nanorods with lengths ranging from 119 to 254.7 nm and widths of approximately 76.521 nm. In contrast, the co-assembly of PTX and indocyanine green (ICG) forms stable nanospheres (diameter: 112 ± 1.06 nm, PDI: 0.1) [43,50,51].

**Figure 4:**
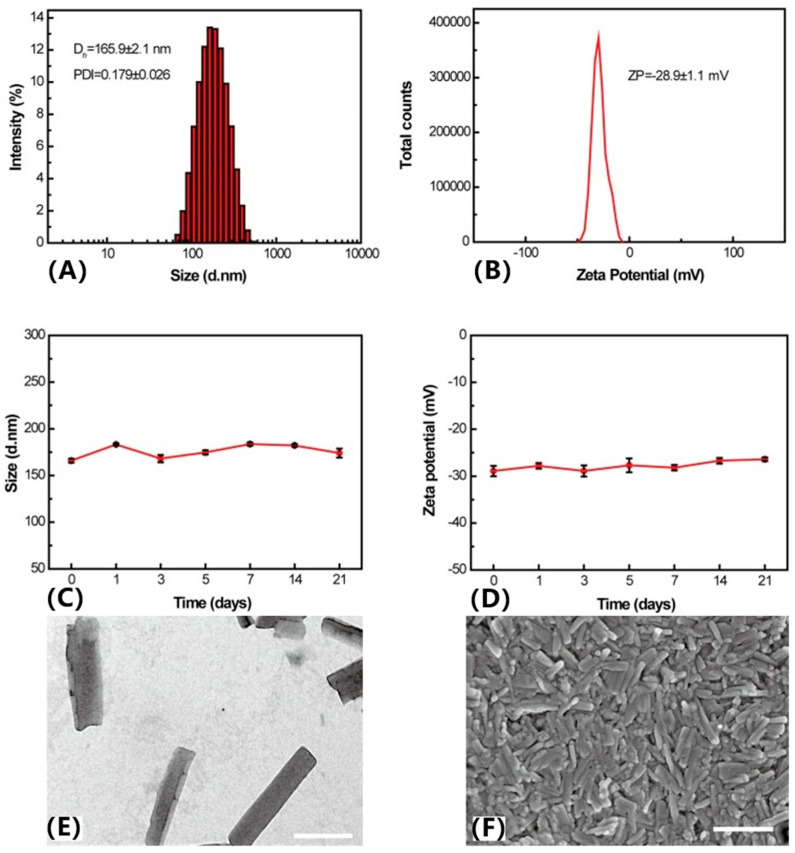
Distribution of (**A**) particle size and (**B**) zeta potential; stability of (**C**) particle size and (**D**) zeta potential as a function of time (0 to 21 days) in purified water. Dh, mean hydrodynamic diameter. TEM images of (**E**) HCPT/Ce6 NRs (molar ratio HCPT: Ce6 = 3:1). SEM images of (**F**) HCPT/Ce6 NRs [44].

The ratio of drug molecules can also impact the morphology and stability of the nano-assemblies. Zhao et al. developed novel carrier-free dual-functional HCPT/Ce6 NRs using different molar ratios of HCPT to Ce6 (1:1, 2:1, 3:1, and 4:1) to determine the optimal formulation [44]. Results indicated that NRs with a 3:1 molar ratio had the smallest particle size (165.9 ± 2.1 nm) and a relatively narrow size distribution (PDI = 0.179 ± 0.026) compared to other ratios. As the HCPT/Ce6 ratio increased, particle size decreased and size distribution narrowed. Combining ITC and storage stability results, the 3:1 ratio of HCPT/Ce6 NRs emerged as the optimal formulation. A suitable particle size of less than 200 nm aids nanoparticles in evading reticuloendothelial system (RES) uptake and achieving passive tumor targeting based on the enhanced permeability and retention (EPR) effect [52]. Additionally, the zeta potential of HCPT/Ce6 NRs (3:1) was −28.9 ± 1.1 mV, indicating minimal hemolysis and cytotoxicity. HCPT/Ce6 NRs exhibited uniform morphology and excellent stability in aqueous and lyophilized states (Figure 4). Upon laser irradiation, HCPT/Ce6 NRs demonstrated substantial cellular uptake efficiency and significant ROS generation capacity both extracellularly and intracellularly. Furthermore, in vitro and in vivo antitumor studies revealed that dual-functional HCPT/Ce6 NRs exhibited remarkable synergistic antitumor efficacy compared to monotherapy with chemotherapy or photodynamic therapy.

**Figure 5:**
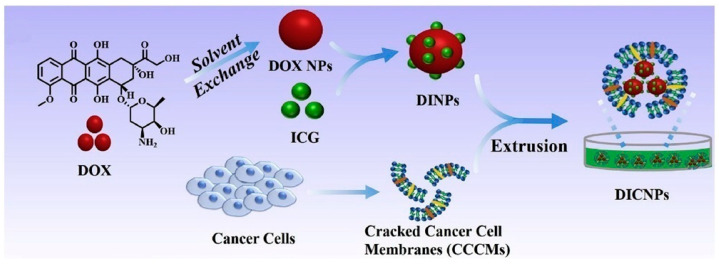
Preparation of cracked cancer cell membranes (CCCMs) and NIR-responsive carrier-free nanosystems (DICNPs) based on packing DOX and ICG co-assembly nanoparticles with CCCMs [45].

Self-assembling small-molecule anticancer drugs and photosensitizers (PSs) into nanomedicines is a straightforward strategy. Loading PSs onto DOX nanoparticles is a more effective method for controlling drug loading efficiency and enhancing cellular uptake, as the rough surface of the nanoparticles facilitates endocytosis by tumor cells [53]. Liu et al. designed a highly efficient carrier-free nanomedicine system based on DOX nanoparticles and ICG [45]. As shown in (Figure 5), DOX nanoparticles were prepared using the reprecipitation method, followed by the assembly of ICG on the surface to form DINP. Notably, the DOX nanoparticles achieved a loading capacity of 89.8%. An HeLa cell-derived cancer cell membrane (CCCM) was then coated on DINP to form DICNPs, protecting DOX and ICG from leakage during blood circulation. The unique homology and targeting capabilities of these nanocarriers facilitated selective accumulation in tumor tissues. Near-infrared light-induced ICG-generated heat disrupted the cell membrane, enabling rapid DOX transfer into cancer cells.

### 3.3. Multidrug Co-Assembly

**Figure 6:**
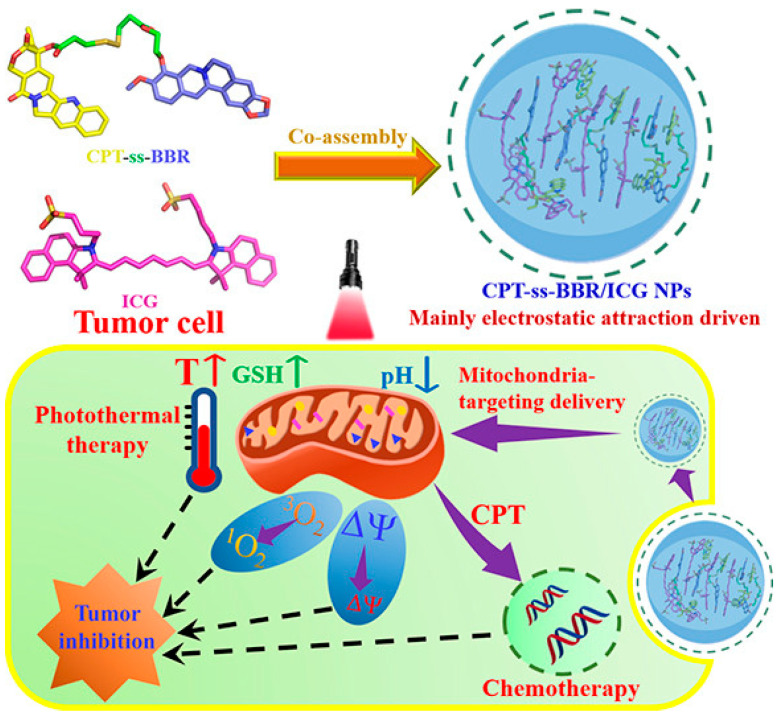
Schematic illustration of the preparation of NIR/GSH/pH-sensitive CPT-ss-BBR/ICG NPs and the chemo-photothermal synergistic therapy process of nanodrugs [46].

In addition to dual-drug co-assembly, researchers have explored the incorporation of multiple small-molecule therapeutics into a single nanosystem to construct multifunctional nanoplatforms. Compared to nanostructures containing one or two drugs, multidrug co-assembly systems exhibit a broader range of characteristics and functionalities. It is well known that Camptothecin (CPT) is a DNA topoisomerase I inhibitor widely used in the clinical treatment of cervical cancer, ovarian cancer, and small-cell lung cancer. Berberine (BBR), with its amphiphilic delocalized positive charge structure, can enter cancer cell mitochondria and induce apoptosis. Cheng et al. co-assembled the CPT and BBR heterodimeric prodrug (CPT-SS-BBR) with the photosensitizer ICG to form a nanostructure (CPT-SS-BBR/ICG NPs), which had an average size of approximately 168 nm (Figure 6) [46]. Computational simulations suggested that the spontaneous binding forces of the co-assembled nanosystem stemmed from hydrophobic interactions, π-π stacking, and particularly the electrostatic interactions between the anionic ICG and the cationic CPT-SS-BBR. Due to the strong electrostatic interactions between the positively charged CPT-SS-BBR and the negatively charged ICG, the CPT-SS-BBR/ICG NPs exhibited excellent stability, remaining stable in phosphate-buffered saline containing 10% fetal bovine serum for up to 20 days. The CPT-SS-BBR/ICG NPs possess the characteristics of lipid cations, enabling them to specifically target the mitochondria of tumor cells. Under the triple stimuli of light (808 nm), acidic conditions, and high concentrations of glutathione (GSH), these nanoparticles rapidly release their drug payloads, accelerating tumor cell apoptosis. Consequently, compared to CPT alone, the multidrug co-assembled nanomedicine exhibited a potent inhibitory effect on A549 cells in the presence of light.

In addition to imaging-guided tumor therapy, multidrug co-assembly systems can be applied to other multimodal treatments. Studies have confirmed that mitoxantrone interacts with DNA to inhibit transcription and translation, paclitaxel suppresses microtubule formation, and 10-hydroxycamptothecin inhibits topoisomerase. Zhou et al. co-assembled these three hydrophobic chemotherapeutic agents, each with distinct anticancer mechanisms, into a single nanorod (MDNCs), which was then modified with polyethylene glycol (PEG) to enhance its dispersibility in water and stability in biological environments [47]. After intravenous injection, the PEGylated MDNCs exhibited a blood circulation half-life of approximately 3 h and demonstrated high accumulation in tumors in vivo due to the enhanced permeability and retention (EPR) effect. This significant accumulation led to a pronounced synergistic effect, enhancing cancer treatment efficacy and imaging capabilities. Both in vitro and in vivo studies highlighted the substantial potential of multidrug co-assembly systems in achieving efficient synergistic chemotherapy, overcoming drug resistance, and improving cancer diagnosis applications.

## 4. Efficient Combination Cancer Therapy Based on Pure Drug Nano-Assemblies

Due to tumor heterogeneity, single-drug therapies are often insufficient to eradicate cancer [54]. Additionally, the development of multidrug resistance (MDR) following the repeated use of a single chemotherapeutic agent can increase the likelihood of tumor metastasis or recurrence after conventional treatments [55]. Therefore, combination therapies involving multiple chemotherapeutic agents or treatment modalities are considered a more promising approach for cancer treatment. As previously discussed, carrier-free nanodrugs have the potential advantage of easily integrating multiple therapeutic agents into a single nanoplatform, facilitating synergistic effects for combination cancer therapy. In the following sections, we will highlight representative applications of carrier-free nanodrugs in combination cancer therapy.

### 4.1. Cancer Theranostics

Theranostics combines diagnostics and therapeutics, providing a comprehensive approach that integrates treatment, diagnosis, and monitoring through imaging. Nano-assemblies, with their exceptional physicochemical properties—such as nanoscale size, functional versatility, active or passive tumor targeting, specific cellular uptake, and excellent optical characteristics—are ideal candidates for theranostic agent carriers [56]. These properties allow nano-assemblies to simultaneously fulfill the requirements for both phototherapy and imaging. The field of nanomaterial-based cancer theranostics holds significant promise, drawing substantial interest from researchers in the development of theranostic agents.

Li et al. reported a layered self-assembly system based on p-aminophenol and diethylenetriamine, possessing self-driving and energy conversion capabilities [57]. This system is specifically activated by β-galactosidase (β-Gal), dynamically forming assemblies with excellent photothermal and fluorescent properties in situ, enabling the multi-modal detection of β-Gal activity. Due to the overexpression of β-Gal in ovarian cancer cells, the system specifically generates self-assembled supramolecules in SKOV-3 cells, facilitating fluorescent imaging. The photothermal therapeutic capability of the synthesized self-assembled oligomers was evaluated in a subcutaneous ovarian cancer model, demonstrating satisfactory anti-tumor effects.

**Figure 7:**
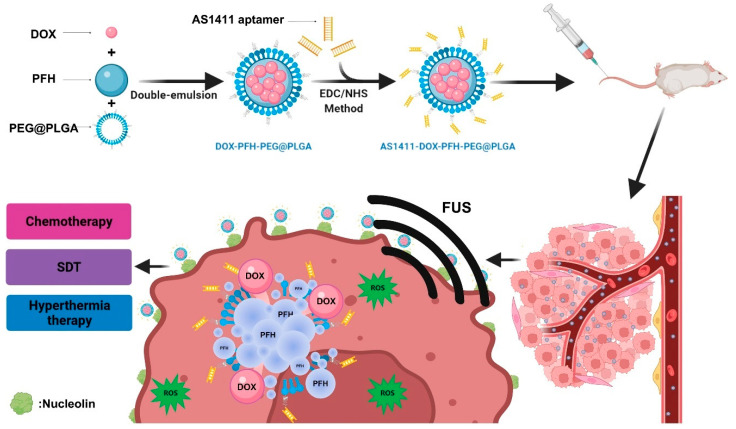
Schematic diagram of enhanced tumor-targeted imaging and synergistic FUS ablation and chemotherapy using nano therapeutic diagnostic agent A-DPP [58].

Kang et al. developed a novel theranostic agent, AS1411-DOX/PFH-PEG@PLGA nanoparticles (A-DPPs) [58]. These nanoparticles exhibit excellent tumor-targeting ultrasound imaging properties due to the phase transition of PFH and pH/ultrasound dual-responsive release characteristics, enhancing both chemotherapy under focused ultrasound (FUS) exposure and ultrasound-induced hyperthermia (Figure 7). Furthermore, the synergistic effects of DOX chemotherapy and FUS treatment, along with the sonodynamic therapy (SDT) effect, overcome the limitations of monotherapy and improve therapeutic efficacy against triple-negative breast cancer. The potential mechanism of this synergy is that the combined FUS and A-DPP treatment enhances ultrasonic cavitation and thermal effects, effectively triggering biological responses.

### 4.2. Combined Chemotherapy

In clinical practice, combining chemotherapeutic agents with different mechanisms often achieves synergistic therapeutic effects. However, the varying physicochemical properties and pharmacokinetics of these drugs can hinder their synergistic efficacy [59]. Self-assembling two or more chemotherapeutic agents into pure drug nano-delivery systems not only ensures a uniform delivery process, achieving synergy, but also significantly enhances drug loading capacity and delivery efficiency.

Xiao et al. developed carrier-free nanoparticles containing two drugs for synergistic combination chemotherapy to overcome DOX resistance. They co-assembled two clinical drugs, celastrol (CST) and DOX, into a nanostructure (CST/DOX NP) [60]. In this nanoparticle, CST inhibits the activation of P-glycoprotein (P-gp), effectively reducing drug efflux, resulting in DOX accumulation approximately four times higher than that of free drugs. This co-assembly achieves synergistic chemotherapy, increasing apoptosis and autophagy rates in multidrug-resistant (MDR) tumor cells. Compared to free DOX, free CST, and the CST/DOX mixture, CST/DOX nanoparticles activate heat shock factor 1 (HSF-1), inhibit P-gp expression, and promote HSF-1 nuclear translocation. Furthermore, CST/DOX nanoparticles inhibit NF-κB through the ROS/κ signaling pathway, inducing apoptosis and autophagy in resistant tumor cells (MCF7/ADR cells), thereby achieving synergistic chemotherapy.

**Figure 8:**
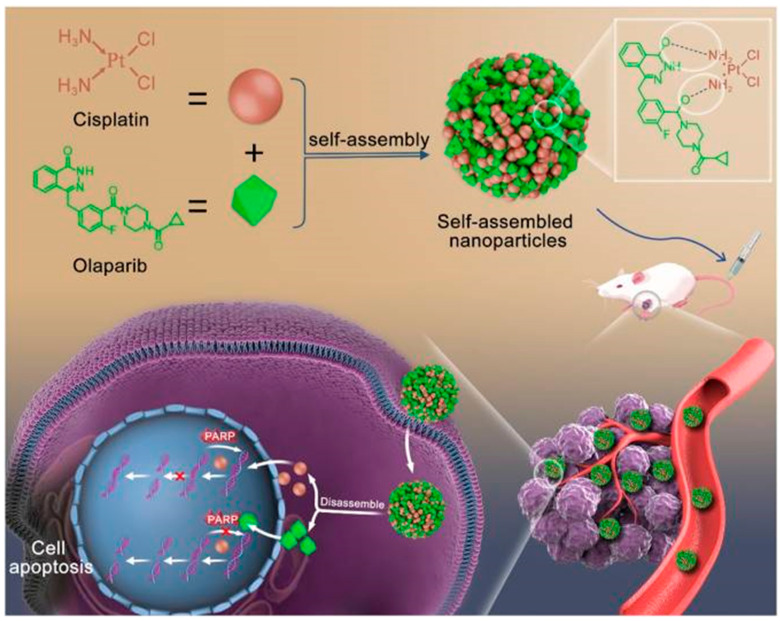
Schematic diagram of self-assembly route of CDDP-OLA nanoparticles and their synergistic mechanism [62].

Cisplatin is a widely used chemotherapeutic agent that induces DNA damage and promotes cancer cell apoptosis. However, intracellular PARP1 repairs damaged DNA strands, reducing the anticancer efficacy of cisplatin. Olaparib, a PARP inhibitor, prevents DNA repair, thereby enhancing cisplatin chemotherapy sensitivity [61]. Nonetheless, both drugs suffer from poor water solubility and limited tumor-targeting capabilities. Thus, Zhang et al. addressed these issues by co-assembling the two drugs into stable and uniform nanoparticles through hydrogen bonding (Figure 8) [62]. This self-assembled system effectively targets tumor cells and releases cisplatin and olaparib locally in the acidic tumor microenvironment due to hydrogen bond cleavage, achieving a synergistic antitumor effect. This approach significantly enhances drug solubility, tumor targeting, bioavailability, and synergistic anticancer efficacy while minimizing the toxic side effects of both drugs.

### 4.3. Combination Phototherapy

Phototherapy has been widely explored in the anti-tumor treatment process due to its significant efficacy and non-invasive advantages. The most representative approaches are photothermal therapy (PTT) and photodynamic therapy (PDT). PTT utilizes strong light absorption and the high photothermal conversion efficiency (PCE) of photothermal transduction agents (PTAs) to absorb light energy and efficiently convert it into heat energy. This raises the local tumor temperature, leading to the rupture of tumor cell membranes or protein denaturation, inducing tumor cell death. The local high temperature also induces various changes in tumor cells, such as triggering the release of antigens from dying tumor cells induced by PTT, promoting the activation of anti-tumor immunity [63]. Additionally, PTT generates excessive reactive oxygen species (ROS) under near-infrared irradiation, causing damage to DNA, proteins, and lipids, ultimately leading to the apoptosis of cancer cells.

To achieve effective PTT, the key factor is the photothermal conversion agent (PTA). Currently, nanomaterials such as gold nanoparticles and nanorods, graphene and graphene oxide, and light-absorbing polymers like polypyrrole have commonly been used as photosensitizers [64]. However, inorganic photothermal materials have long been constrained by their non-biodegradability and non-biological derivatives, limiting their further clinical application due to safety concerns. While the emergence of polymer nanoparticles has overcome some of the drawbacks of inorganic photothermal materials, many of them still face limitations, including complex manufacturing processes, unclear biodegradability, and potential biosafety issues. As an alternative and innovative strategy, biologically sourced simple biomolecules provide an answer to the aforementioned challenges in photothermal material design. Zou and colleagues developed a supramolecular strategy, manufacturing photothermal nanodots through the peptide-regulated self-assembly of photosensitive porphyrins [64]. The self-assembly property of porphyrins induced the formation of J-aggregates as substructures of nanodots, thereby enabling the creation of nanodots that completely suppress fluorescence emission and generate singlet oxygen, achieving high photothermal conversion efficiency. The assembled peptide–porphyrin photothermal nanodots exhibit advantages of high tumor accumulation and low toxicity while maintaining long-term colloidal stability and blood circulation stability.

PDT is a process in which photosensitizers (PSs) convert oxygen (O_2_) into cytotoxic reactive oxygen species (ROS) under light exposure, directly triggering intrinsic mitochondrial oxidative damage-related apoptotic pathways, thereby achieving the purpose of killing tumor cells. The main characteristics of PDT are its strong selectivity and minimal invasiveness. In addition to destroying tumor cells through ROS, PDT can even induce immunogenic cell death (ICD), accompanied by the generation and release of damage-associated molecular patterns (DAMPs), which are recognized by various immune cells, thereby eliciting a specific anti-tumor immune response [65].

Photosensitizers are the most critical elements in PDT, playing a decisive role in its effectiveness. BODIPY dyes are well-established fluorescent dyes; however, their poor water solubility, low tumor selectivity, and limited biocompatibility hinder their biological applications [66]. Supramolecular assembly strategies offer a promising approach to overcoming the current limitations of BODIPY-based molecular photosensitizers in PDT for cancer treatment. For instance, Liu et al. reported that BODIPY-based photosensitizers can spontaneously self-assemble into stable nanoparticles in water, producing O_2_ at a rate 24–124 times higher than the corresponding molecules in organic solutions [67]. Photodynamic experiments demonstrated that under 660 nm wavelength light, the nanoparticles could also target the mitochondria of cancer cells and induce apoptosis by decreasing the mitochondrial membrane potential.

**Figure 9:**
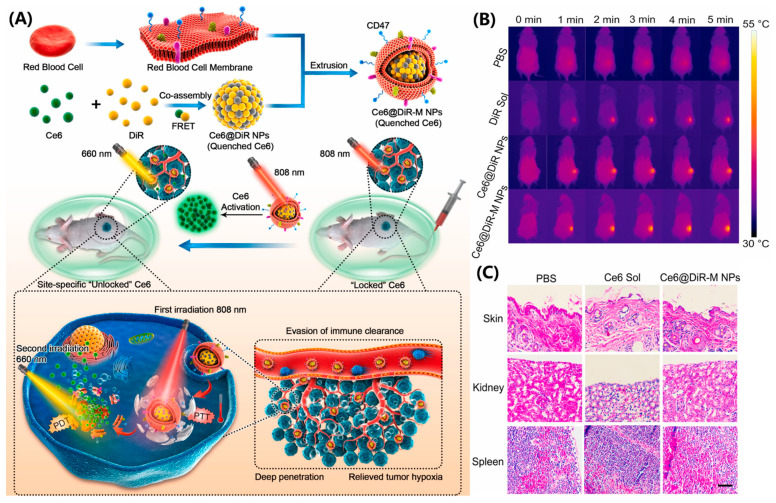
(**A**) Schematic representation of the preparation of erythrocyte camouflaged Ce6@DiR NPs (Ce6@DiR-M NPs) and its programmed cascade-activatable photothermal–photodynamic therapy for TNBCs with low phototoxicity in normal tissues. (**B**) In vivo photothermal images under 808 nm laser irradiation (2 W cm^−2^, 5 min). (**C**) H&E stain of skin, spleen, and kidney after the 660 nm laser (5 mW cm^−2^ for 1 h) treatment (scale bar represents 50 μm) [69].

Furthermore, the combined treatment of PDT and PTT is ideal, as PDT can increase tumor cell sensitivity to PTT by interfering with the tumor microenvironment (TME). The heat generated by PTT can increase blood flow, thereby improving O_2_ supply and enhancing PDT efficacy [68]. Despite the promising prospects of PTT/PDT combination therapy, severe phototoxicity still brings significant suffering and inconvenience to cancer patients. Nanoparticle assemblies driven purely by drugs can achieve controlled phototoxicity for efficient combined therapy, addressing this challenge. Zhang et al. developed red blood cell camouflaged nanoparticle assemblies of Ce6 and DiR (Ce6@DiR-MNP) for the treatment of triple-negative breast cancer (Figure 9A) [69]. The red blood cell coating not only stabilizes Ce6@DiR nanoparticles but also improves the pharmacokinetic behavior for multiple administrations. In Ce6@DiR-MNP, Ce6 is quenched by DiR and can only be released for PDT when DiR is irradiated with an 808 nm laser, thereby effectively controlling the phototoxicity induced by Ce6. Under cascade laser irradiation (660–808 nm), the temperature increase induced by DiR in tumor localization not only triggers tumor cell apoptosis but also promotes nanoparticle penetration into the tumor, alleviating hypoxia and enhancing the PDT efficacy of Ce6 (Figure 9B). Ce6@DiR-MNP, as a multifunctional co-delivery nanoplatform, demonstrates synergistic phototherapy and high safety (Figure 9C).

### 4.4. Chemotherapy Combined with Phototherapy

The combination of chemotherapy and phototherapy, specifically the carrier-free chemo-photothermal therapy, integrates the benefits of both treatment modalities. This approach enhances the pharmacodynamics and pharmacokinetics of chemotherapeutic drugs, allows for high drug loading, and reduces toxicity, making it a promising strategy for improving anticancer efficacy. Despite its therapeutic advantages, achieving satisfactory results with chemo-phototherapy remains challenging, primarily due to the asynchronous pharmacokinetic behaviors and tumor accumulation profiles of chemotherapeutic drugs and phototherapeutic agents [70]. Therefore, the rational design of more efficient drug co-delivery systems is crucial for effective chemo-phototherapy.

Gambogic acid (GA), a naturally derived chemotherapeutic agent, has garnered increasing attention in antitumor therapy. However, current research mainly focuses on improving its pharmacological properties to overcome clinical application limitations or as a synergistic anticancer drug in combination with chemotherapy and chemo-phototherapy, often overlooking its self-assembly material properties. Lin et al. validated the self-assembly capability of GA using pyropheophorbide-a (PPa) as a model drug, demonstrating its significant potential as a single-component active carrier for synergistic delivery [71]. The GA/PPa combination exhibited markedly improved GA water solubility and several advantageous therapeutic properties, achieving an in vivo treatment efficiency of up to 89.3% while reducing GA hepatotoxicity.

**Figure 10:**
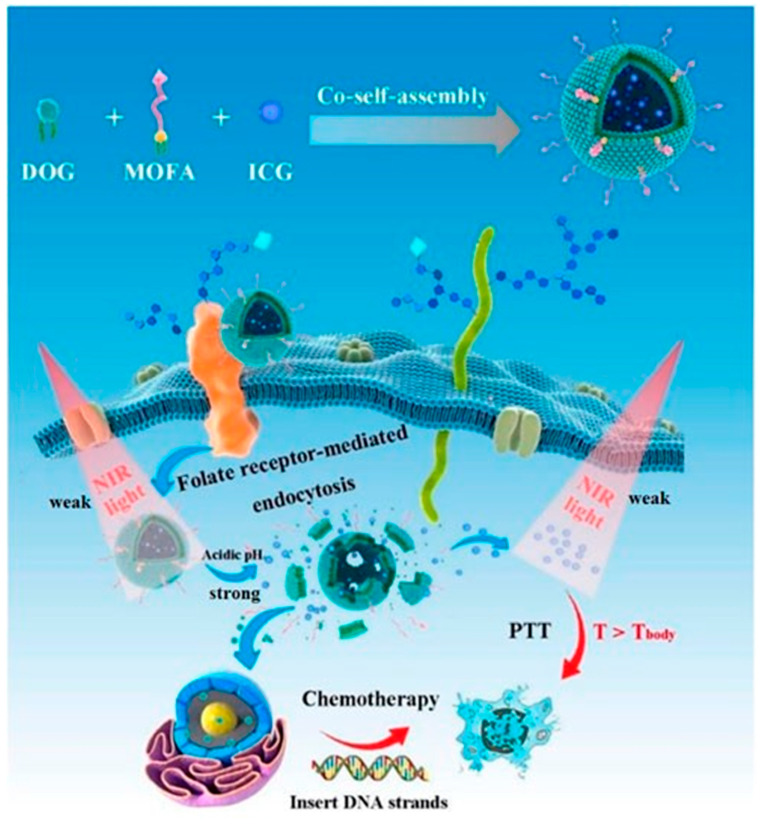
Schematic diagram of the preparation of DOG/MOFA/ICG nanoparticles and their action [72].

Kuang et al. designed novel carrier-free targeted nanoparticles for synergistic chemo-photothermal therapy, consisting of the amphiphilic prodrug 3′,5′-dioleoyl gemcitabine (DOG) and tumor-targeting γ-octadecyl folic acid (MOFA), co-assembled with the photosensitizer indocyanine green (ICG) (Figure 10) [72]. These nanoparticles presented a monodisperse nanoscale structure with a high ICG encapsulation efficiency (approximately 74%). The system demonstrated acid- and laser-triggered GEM release in vitro, sustained GEM release in vivo following intravenous injection, and excellent temperature conversion capability (57.0 °C). Compared to standalone chemotherapy or photothermal therapy, the combination of DOG/MOFA/ICG nanoparticles with near-infrared laser irradiation exhibited superior antitumor efficacy, with extremely low hemolysis rates and minimal toxicity to L929 cells.

### 4.5. Combination of Immunotherapy with Other Therapies

Tumor immunotherapy, which has rapidly emerged as a promising cancer treatment, has garnered significant attention due to its high specificity and minimal side effects, achieving notable success [73]. The primary strategy of tumor immunotherapy involves immune checkpoint inhibitors, which induce or enhance T-cell-mediated antitumor responses, leading to immunogenic cell death. However, traditional immune checkpoint blockade therapy faces challenges such as low immunogenicity, weak targeting, resistance development, and cytokine storms, which hinder its clinical translation [74]. Self-assembled nanoparticles can alter the distribution behavior of drugs, enhance targeting effects, achieve precise control, and enable drug accumulation at tumor sites, thereby increasing local drug concentration and reducing off-target effects while continuously mitigating immunotoxicity.

Phototherapy can act as a precursor and enhancer of antitumor immunity by inducing immunogenic cell death. However, single-mode phototherapy has limited efficacy in achieving long-term and systemic antitumor immune responses. When combined with immunotherapy, phototherapy can efficiently kill tumor cells, enhance immune cell infiltration into tumors, and modulate the TME from anti-inflammatory to pro-inflammatory pathways, thus improving tumor eradication. This combination therapy triggers effective coordination between innate and adaptive immunity, targeting not only primary tumor cells but also distant metastatic cells [75].

**Figure 11:**
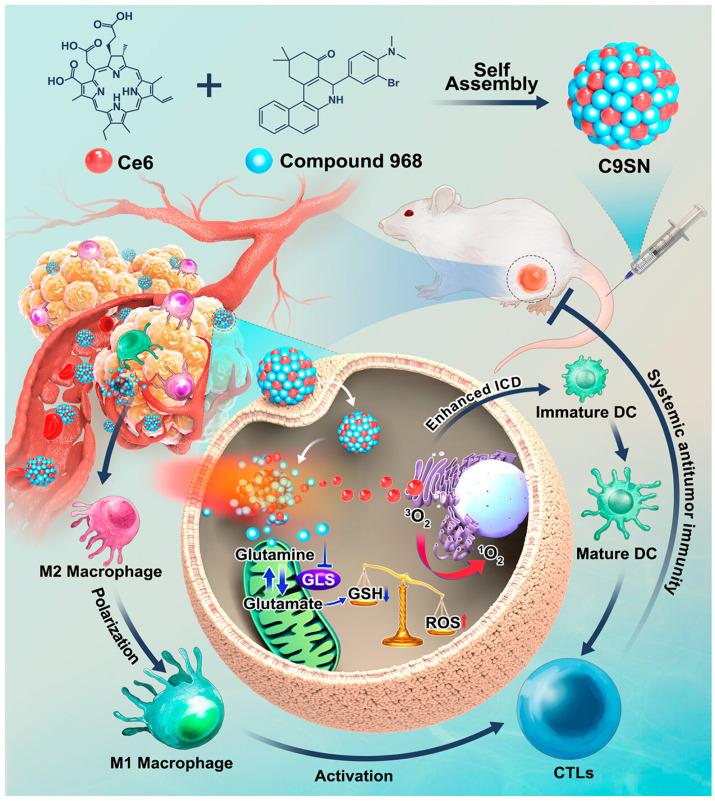
Schematic illustration of antitumor synergistic immunotherapy mediated by C9SN with laser irradiation via increasing tumor immunogenicity and reversing immunosuppressive tumor microenvironment [76].

Mai et al. developed a carrier-free immunotherapy nano-enhancer, C9SN, with dual synergistic effects through the self-assembly of the glutaminase (GLS) inhibitor compounds C968 and Ce6 [76]. C968 amplifies intracellular oxidative stress, leading to severe cell death and enhanced ICD effects (Figure 11). The enhanced ICD effect generates new antigens that promote dendritic cell maturation, recruiting and activating cytotoxic T lymphocytes (CTLs). Additionally, C9SN can reprogram the TME by polarizing M2-type tumor-associated macrophages to M1-type, further recruiting and activating CTLs, thereby inhibiting both primary and distant tumors.

**Figure 12:**
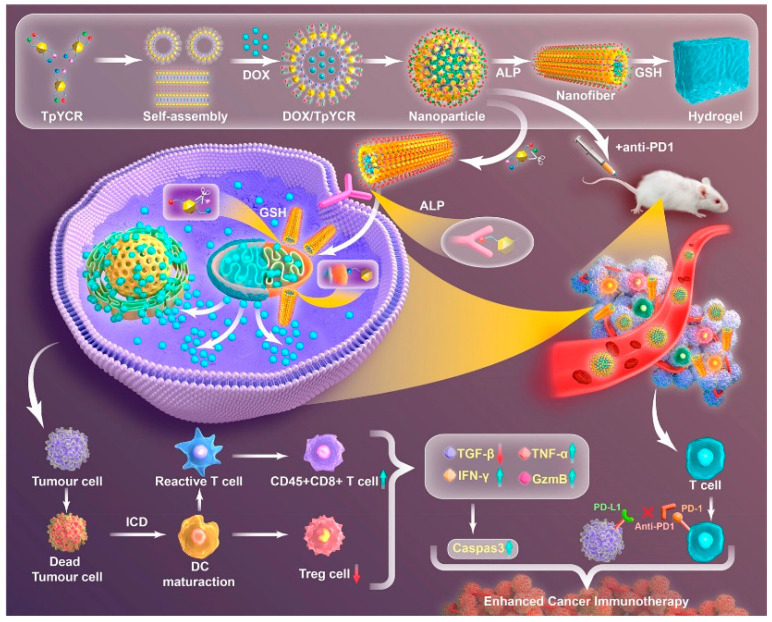
Construction of a self-assembled peptide, TpYCR, for stepwise targeting and tandem response-triggered morphological transition [78].

Similarly, integrating chemotherapy into immunotherapy enhances tumor control. Chemotherapy effectively suppresses immunosuppressive cells, promotes immune responses by inducing tumor cell apoptosis, and matures dendritic cells (DCs) [77]. Therefore, the organic combination of chemotherapy and immunotherapy holds promise for the precise treatment of malignant tumors. Cao et al. developed a transition-enhanced immunomodulation (TEI) effect to construct self-assembled peptide TpYCR for the sequential targeting of tumor cells and mitochondrial drug delivery [78]. DOX/TpYCR first targets tumor cells, where alkaline phosphatase (ALP) in the tumor microenvironment induces the transformation from nanoparticles (NPs) to nanofibers (NFs), releasing DOX. Subsequently, the intracellular overexpression of reduced glutathione (GSH) induces hydrogel formation, increasing the intracellular retention of DOX and continuously inducing ICD. Importantly, DOX/TpYCR inhibits Treg cells, activates DC cells, and stimulates the expression of immunogenic factors, generating a lasting immune response. The combination of PD-1 inhibitors with chemotherapy further enhances the response rate of immune inhibitors, producing a synergistic effect that ultimately inhibits tumor growth and metastasis (Figure 12).

**Figure 13:**
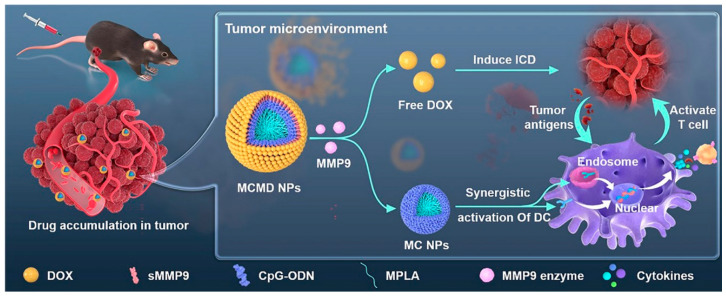
Schematic illustration of the synergistic anti-tumor chemo-immunotherapeutic mechanism of carrier-free MCMD nanoparticles [80].

Nucleic acid drugs, including DNA and RNA, play a crucial role in cancer therapy by regulating gene transcription and expression. These drugs have emerged as one of the most promising categories in biopharmaceutical research and development. The combination of nucleic acid drugs with immunotherapy, a popular treatment strategy in recent years, has attracted significant attention across various fields [79]. However, nucleic acid drugs face major challenges such as poor stability, weak targeting in vivo, and difficulty in overcoming physiological barriers. The selection of nanostructures for gene and immunotherapy combination is key to achieving high therapeutic efficacy. Ma et al. developed carrier-free core–shell nanoparticles (MPLA-CpG-sMMP9-DOX, MCMD NPs) through a simple self-assembly of spherical nucleic acids (SNAs) using CpG ODN and monophosphoryl lipid A (MPLA) as adjuvants and DOX as the shell [80]. The results demonstrated that MCMD NPs enhance drug accumulation in tumors and release DOX after the enzymatic degradation of matrix metalloproteinase-9 (MMP9) in the TME, thereby enhancing the direct cytotoxic effect of DOX on tumor cells (Figure 13). The MPLA-CpG SNA core effectively boosts the antitumor immune response induced by ICD, further targeting and destroying tumor cells.

## 5. Conclusions

This article provides a comprehensive review of recent advancements in the application of PDANSs in cancer treatment. It examines the underlying self-assembly mechanisms and categorizes self-assembly strategies based on drug composition. The discussion delves into combination treatment strategies enabled by PDANSs, including the self-assembly of chemotherapeutic agents to enhance drug delivery efficiency, the self-assembly of phototherapy agents to mitigate phototoxicity, and the integration of chemotherapeutic and phototherapeutic drugs within PDANSs to achieve synergistic therapeutic effects (“1 + 1 > 2”). Additionally, it explores the combination of immunotherapy with other treatment modalities to achieve enhanced therapeutic outcomes. Finally, the article evaluates the potential applications of PDANSs in tumor therapy and their prospects for clinical translation, offering valuable insights to guide future research aimed at optimizing PDANSs and expanding their use in anticancer therapies.

### 5.1. Challenges

PDANSs have been widely applied in biomedicine, addressing issues such as the low solubility of small-molecule drugs, poor tumor targeting, significant side effects, and strong drug resistance [clinical applications of nanomedicine examples (Table 3)]. However, the development and clinical translation of this technology still face several challenges.

First, although PDANSs can co-deliver multiple drugs, optimizing their ratios to ensure stability and maximize synergistic therapeutic effects remains a significant challenge. Second, predicting the self-assembly behavior and morphology of carrier-free nanomedicines is complicated by the lack of a clear understanding of the assembly forces and mechanisms. This unpredictability can impact the stability and efficacy of PDANSs in vivo. Third, the clinical translation of PDANSs faces regulatory hurdles, as their unique pharmacokinetics and reduced toxicity require the development of novel methods for drug quality control and analysis. Traditional toxicological evaluation protocols may also need to be adapted to assess the safety of these systems effectively. Moreover, scaling up the production of PDANSs from laboratory research to industrial manufacturing poses additional difficulties. This process demands extensive optimization to ensure stable raw material supply, robust quality control measures, and the consistent maintenance of physicochemical properties and biological activity across different production scales. Finally, the most crucial question is whether PDANSs will be as effective in human trials as they have been in preclinical studies [81]. Despite the long road to clinical translation, PDANSs demonstrate a promising advanced combination therapy.

### 5.2. Outlook

PDANSs, with their functional modifications and transformation potential, have been extensively used in tumor diagnosis, chemotherapy, phototherapy, and immunotherapy combinations. They offer several advantages, including simple and repeatable preparation techniques, ultra-high drug loading capacity, and efficient co-delivery behavior, making them a promising option for clinical oncology. With continued advancements in drug development, emerging technologies such as machine learning and artificial intelligence are playing an increasingly crucial role in improving drug design and optimization. These technologies enable the efficient analysis of large datasets, the identification of promising drug candidates, the prediction of the self-assembly behaviors of drug molecules, and the optimization of drug loading capacities and assembly morphologies. Looking ahead, it is expected that PDANSs will advance to clinical trials and ultimately establish themselves as viable medical products, paving the way for innovative and effective therapeutic solutions.

## Figures and Tables

**Table 1:** Advantages and disadvantages of traditional nanodrug delivery systems and PDANSs.

	Advantages	Disadvantages
Nanocarrier delivery system	Good drug stability Controlled release Surface modification for targeting	Complicated preparation process Low drug loading efficiency (<10%) Potential nanomaterial toxicity
PDANS	Simple preparation process High drug loading (close to 100%) Non-toxic carriers with good biocompatibility Smaller particles for better penetration and tissue retention	Poor stability (simple structure, unstable in vivo) Inadequate targeting

**Table 2:** Three PDANS types for cancer therapy.

Types	Drug 1	Drug 2	Drug 3	Drug Loading	Reference
Single pure drug nano-assemblies	Pyropheophorbide a	-	-	74.8%	[41]
	Dihydroartemisinin	-	-	>92%	[42]
Dual pure drug nano-assemblies	Indocyanine green	Paclitaxel	-	≈100%	[43]
	10-Hydroxycamptothecin	Chlorin e6	-	-	[44]
	Indocyanine green	Doxorubicin	-	89.8%	[45]
Multi-pure drug nano-assemblies	Indocyanine green	Berberine	Camptothecin	-	[46]
	10-Hydroxycamptothecin	Methotrexate	Paclitaxel	100%	[47]

**Table 3:** Nanomedicines in clinical trials for cancer therapy.

Trade Name	Nanotechnology Platform	Cancer Type	First Approval
Doxil	PEGylated Doxorubicin Liposomes	Ovarian Cancer, Kaposi’s Sarcoma, Breast Cancer, Multiple Myeloma	USA
DaunoXome	Doxorubicin Liposome	Kaposi’s Sarcoma	USA
Marqibo	Vincristine Liposomal	Leukemia	USA
Myocet	Doxorubicin Liposome	Breast Cancer	Europe
MEPACT	Muramyl Tripeptide Phosphatidylethanolamine Liposomes	Osteosarcoma	Europe
SMANCS	Polymer-based Novel Anticancer Drug Conjugates	Hepatocellular Carcinoma, Renal cell Carcinoma	Japan
Genexol-PM	Paclitaxel Micelles	Breast Cancer, Small-Cell Lung Cancer	Korea
Lipusu	Paclitaxel Liposome	Breast Cancer, Lung Cancer, Ovarian Cancer	China
DepoCyt	Cytarabine Liposome	Lymphoma Meningitis	USA
Abraxane	Albumin-bound Paclitaxel Nanoparticles	Multiple Cancers, Metastatic Pancreatic Cancer	USA
Oncaspar	Polymer–Protein Conjugate	Acute Lymphoblastic Leukemia	USA
Eligard	Leuprorelin Acetate Polymer	Prostate Cancer	USA
Ontak	Leuprorelin Acetate Polymer	Cutaneous T-Cell Lymphoma	USA
Vyxeos	Cytarabine and Daunorubicin Liposomes	Acute Myeloid Leukemia	USA
Nano-therm	Iron Oxide Nanoparticles	Glioblastoma	USA
Onivyde	Irinotecan Liposome	Pancreatic Cancer	USA
Nanoxel	Paclitaxel Polymeric Micelles	Multiple Cancers	India
Paclical	Paclitaxel Micelles	Ovarian Cancer, Fallopian Tube Carcinoma, Peritoneal Cancer	Russia
Hensify	Hafnium Oxide Nanoparticles	Soft-Tissue Sarcoma	Europe
Liporaxel	Paclitaxel Liposome	Gastric Cancer	Korea
PICN	PICN	Breast Cancer	India
Duoenda	Mitoxantrone Liposome	Peripheral T-Cell Lymphoma	China
